# TMD and Bruxism as Expressions of Central Regulatory State: The Functional Occlusion Regulated Model (FORM)

**DOI:** 10.3390/jcm15124567

**Published:** 2026-06-12

**Authors:** David Cheng

**Affiliations:** Westcoast Smile Dental Studio & Whitening Spa, 1-1874 West 1st Avenue, Vancouver, BC V6J 1G5, Canada; toothfixer86@gmail.com

**Keywords:** temporomandibular disorders, bruxism, sleep bruxism, masticatory performance, autonomic nervous system, heart rate variability, central regulation, occlusion, neuromuscular efficiency, resonance frequency breathing

## Abstract

Temporomandibular disorder (TMD) and bruxism affect a significant portion of the adult population, yet why patients with comparable occlusal findings respond so differently to identical interventions remains unexplained by both structural and biopsychosocial frameworks. Traditional occlusal paradigms identified the importance of structural relationships but could not account for clinical variability. Biopsychosocial models advanced understanding of central regulation but lack the physiological specificity needed to connect regulatory state to functional jaw behavior. This paper proposes the Functional Occlusion Regulated Model (FORM), a hierarchical framework integrating central regulatory state, masticatory performance, and structure into a coherent model of jaw function, and identifies its clinical and research implications. Narrative synthesis of the peer-reviewed literature across masticatory physiology, autonomic neuroscience, pain neuroscience, and clinical dentistry was conducted; seventy-two references are cited. Converging evidence supports a three-tier hierarchy in which autonomic and neuromuscular regulatory state is proposed to shape masticatory performance, which influences how structural occlusal conditions are expressed and clinically experienced. FORM generates four testable predictions distinguishing it from existing models, and a preliminary clinical observation documents symptom resolution through regulatory intervention alone without occlusal modification, representing an early published dental observation of this connection. FORM provides a physiologically grounded framework for understanding treatment response variability and proposes central regulatory state as a potentially important upstream influence on functional jaw outcomes.

## 1. Introduction

The observations motivating this framework emerge from 35 years of clinical practice spanning restorative, implant, orthodontic, and occlusal rehabilitation dentistry. That practice was informed by advanced training at the LD Pankey Institute, the Dawson Academy, the Misch Implant Institute, and the Kois Center, alongside dedicated study in autonomic physiology, breathing science, and heart rate variability biofeedback under Peter Litchfield and Maria Katsamanis of the Behavioral Physiology Institute. Across that breadth of clinical and scientific training, the same unexplained pattern repeatedly surfaced: structurally similar patients responding entirely differently to identical interventions.

Two clinical observations crystallized the question this framework is designed to answer. The first involved a patient who developed TMJ symptoms following orthodontic bite pad removal. Examination revealed absent posterior occlusal support across the bicuspids and molars. Transitional composite bonding restored bilateral equal simultaneous contacts across the full posterior occlusal table, resolving the pain—not through structural correction alone, but by restoring masticatory efficiency. The second involved a patient with TMJ pain, sleep bruxism, and chronic headaches. No occlusal modification was made. A structured program of heart rate variability biofeedback and resonance frequency breathing training resolved the symptoms. Two patients. The same clinical endpoint. Entirely different levels of intervention.

No existing framework provides a unified mechanistic account of both outcomes. FORM was developed to address that gap.

Temporomandibular disorder (TMD) and bruxism are among the most common conditions encountered in dental practice, affecting function, comfort, and quality of life across a broad patient population. Epidemiologic studies indicate that clinically significant TMD affects approximately 10% of adults, with a higher prevalence among women [1]. Bruxism is similarly widespread, though prevalence estimates vary substantially depending on age group, diagnostic criteria, and whether assessment is based on self-report or instrumental recording [2]. Despite decades of research and evolving clinical guidelines, these conditions continue to generate diagnostic uncertainty and inconsistent treatment outcomes [3].

Two major explanatory traditions have shaped clinical thinking. The first, rooted in gnathological principles, identifies static occlusal relationships—centric relation contacts, anterior guidance, and occlusal interference—as upstream determinants of jaw function and dysfunction [4,5]. The second, emerging from pain neuroscience and behavioral medicine, reframes TMD as a centrally mediated condition modulated by psychological stress, autonomic arousal, and central sensitization, with occlusion reconsidered as one among several contributing factors rather than the primary upstream determinant [6,7]. Each tradition has generated genuine clinical insights, yet neither fully resolves the variability clinicians observe, why occlusal correction reliably helps some patients and does nothing for others, why bruxism waxes and wanes with stress independently of any dental change, and why masticatory performance can differ dramatically between patients whose tooth contacts appear comparable.

The gap between these models is not merely theoretical. It has practical consequences for clinical decision-making. Without a framework that integrates central regulatory state with peripheral jaw function, clinicians are left choosing between structural correction and psychosocial management as if they were mutually exclusive—when FORM proposes they are dynamically related.

This paper proposes the Functional Occlusion Regulated Model (FORM) as an integrative framework to address this gap. FORM reframes occlusion not as a static structural relationship to be measured and corrected, but as a dynamic functional interface through which centrally regulated states are expressed during mastication. Within FORM, masticatory performance—how efficiently and smoothly an individual chews—emerges as a clinically meaningful indicator of neuromuscular efficiency and autonomic regulation, providing a functional bridge between central state and occlusal behavior. Rather than replacing existing models, FORM integrates and extends them by proposing a physiological specificity that current biopsychosocial frameworks have not yet specified, and a central regulatory context that traditional occlusal theory has not addressed. FORM should be viewed as a physiological extension of existing biopsychosocial concepts rather than a replacement for them. The hierarchy presented in FORM represents a conceptual model derived from converging evidence and is not intended to imply established causal weighting among tiers.

The theoretical foundation of FORM is drawn from converging evidence in mastication research, autonomic physiology, pain neuroscience, and clinical dentistry. The following sections examine the contributions and explanatory boundaries of existing models, present the evidence base underlying FORM, define the model and its core constructs, and discuss its clinical implications across restorative, implant, orthodontic, and occlusal appliance contexts.

### Search Methodology

The literature for this narrative review was identified through searches of PubMed/MEDLINE and Google Scholar, conducted from 1990 through 2025. Search terms were organized across five thematic domains directly relevant to the theoretical framework under development: (1) masticatory physiology, jaw kinematics, and masticatory performance; (2) autonomic nervous system regulation, heart rate variability, and vagal tone; (3) pain neuroscience, central sensitization, and biopsychosocial models of temporomandibular disorders; (4) breathing physiology, CO_2_ homeostasis, and respiratory psychophysiology; and (5) clinical dental outcomes including implant, orthodontic, and restorative dentistry. Within each domain, searches used both controlled vocabulary and free-text terms. Author field searches were used where keyword searches required greater specificity. Reference lists of identified review articles, consensus documents, and foundational texts were searched manually to retrieve the relevant literature not captured by database searches. Only English-language publications were considered.

Sources published from 1990 through 2025 were reviewed, reflecting the period spanning the emergence of biopsychosocial models of TMD through recent advances in autonomic physiology and mastication research. Foundational studies predating this range were retained where they represented the primary or most widely cited source for a given construct. Source types included peer-reviewed original research articles, systematic reviews and meta-analyses, observational human studies, experimental animal investigations, and clinically relevant case reports, alongside textbooks and clinical guidelines used to represent established occlusal and diagnostic paradigms.

Literature selection was based on conceptual relevance to the regulatory, functional, and structural relationships proposed in FORM. Studies were evaluated for their contribution to one or more of the five thematic domains, with preference given to peer-reviewed experimental and observational evidence over narrative commentary. Where contradictory or null findings were identified within a domain, these were considered in the synthesis and addressed within the relevant sections of the manuscript. Evidence was synthesized thematically to identify areas of convergence, explanatory limitation, and ongoing controversy. This process is consistent with narrative theoretical synthesis methodology and is not intended to represent a systematic review or meta-analysis.

## 2. Toward Integration: The Explanatory Space Both Models Leave Unoccupied

### 2.1. Traditional Occlusal Paradigms: Insights and Limitations

The mechanically organized frameworks that dominated twentieth-century dental education provided genuine and lasting clinical insights. Influential authors including Dawson and Okeson emphasized reproducible mandibular positioning, coordinated muscle activity, and smooth excursive movements as the foundations of functional occlusal health [4,5]. These frameworks were not simply about tooth alignment; they integrated joint position, neuromuscular coordination, and occlusal contacts into a coherent diagnostic and therapeutic system. The clinical objective was stability: a masticatory apparatus that could function with minimal strain and predictable load distribution across supporting structures.

Within this framework, occlusal discrepancies were understood as upstream drivers of dysfunction. Altered tooth contacts were assumed to propagate through the masticatory system, increasing muscle activity and joint loading, and ultimately producing pain or parafunctional behaviors. Intervention was therefore directed at correcting the structural relationship, with the expectation that mechanical optimization would restore neuromuscular balance and resolve symptoms.

Clinical experience and research have shown that this assumption applies to some patients but not others. Systematic reviews evaluating associations between occlusal features and TMD have shown that static occlusal morphology alone is insufficient as a primary etiologic explanation, indicating that additional variables are necessary to account for the full clinical picture [8]. Clinically significant symptoms occur in patients with apparently acceptable occlusion, and patients with marked occlusal irregularities may remain entirely asymptomatic. Occlusal correction produces meaningful relief in some patients and limited benefit in others, a variability that meta-analytic evidence confirms and that the mechanical model alone cannot explain [9]. The mechanical model accounts for the structural contribution to this variability but does not address the central regulatory processes that determine how peripheral occlusal inputs are interpreted and expressed.

### 2.2. Biopsychosocial Models: Advances and Remaining Gaps

The emergence of centrally mediated and biopsychosocial frameworks represented a necessary and important corrective. Advances in pain neuroscience demonstrated that, in many patients with TMD, symptom severity correlates poorly with identifiable peripheral tissue pathology and instead reflects altered nociceptive processing and central amplification [6]. Central sensitization—characterized by increased excitability within central nociceptive pathways—has been identified as a key mechanism for pain hypersensitivity and symptom persistence across chronic musculoskeletal pain conditions [10,11]. Neuroimaging findings in TMD populations further support central involvement, demonstrating functional and structural alterations in brain regions associated with pain modulation, affective processing, and sensorimotor integration [12].

Prospective evidence from the OPPERA cohort demonstrated that psychological distress, somatic symptom burden, and pain amplification predict the development of first-onset painful TMD independently of occlusal or structural findings [13]. These observations situate painful TMD within a broader group of chronic overlapping pain conditions characterized by shared central mechanisms and system-level vulnerability [14]. The Diagnostic Criteria for Temporomandibular Disorders (DC/TMD) formalized this biopsychosocial approach by distinguishing physical diagnoses from psychosocial and behavioral dimensions, improving diagnostic reliability and promoting conservative, patient-centered care [15].

Despite these advances, the biopsychosocial framework has important limitations when applied to clinical dentistry. A significant one is a lack of physiological specificity. Psychological and social factors—stress, anxiety, coping, social support—are consistently associated with pain experience, yet the mechanisms connecting these factors to specific physiological and functional outcomes have not always been fully specified, leaving an important mechanistic gap between central influences and their clinical expression [16,17].

A related problem is the structural organization of diagnostic systems. In frameworks such as the DC/TMD, physical diagnoses and psychosocial factors are classified in parallel rather than integrated within a unified physiological model [15]. This parallel structure, while clinically pragmatic, may not fully capture the dynamic interaction between biological and psychological domains through shared physiological systems. Within this structure, occlusion has not yet been reconceptualized within a unified physiological framework that connects structural and regulatory influences. Contemporary guidelines appropriately caution against irreversible occlusal interventions [18,19] yet the question of how peripheral occlusal inputs interact with centrally regulated pain and motor states remains incompletely addressed within current frameworks.

Emphasis on symptom clusters and psychosocial correlates, while valuable, may leave functional performance and physiological resilience undercharacterized as clinical endpoints, a gap that becomes particularly evident when treatment success correlates poorly with pain intensity reduction alone [20]. Moreover, inconsistent measurement of social factors within chronic pain research further complicates biopsychosocial application, with variables such as perceived social support showing mixed associations with pain outcomes across predominantly cross-sectional designs [21]. Barriers including clinician training, health system limitations, and inconsistent use of validated multi-axial protocols also undermine full implementation, particularly in dental settings [22].

The DC/TMD Axis II instruments—including the PHQ-9, GAD-7, and Graded Chronic Pain Scale—represent the field’s own attempt to operationalize psychosocial assessment within a biopsychosocial framework. Despite their validation, uptake in non-specialist dental settings has remained low, driven by training requirements, time constraints, and the practical complexity of psychometric administration in routine care [23]. FORM does not propose to replace these instruments. It proposes that autonomic regulatory state, assessable through end-tidal CO_2_ and heart rate variability, offers a complementary physiological index of the regulatory burden the biopsychosocial model identifies—one accessible to the general dentist at the chair, without questionnaire administration. This is not a competing framework but an attempt to give the biological tier of biopsychosocial thinking a measurable clinical expression.

### 2.3. The Shared Gap

Each tradition captures an important dimension of the clinical problem. Traditional occlusal models account for structural relationships but not for central regulatory state. Biopsychosocial models account for central and psychosocial influences but not for how those influences are expressed through jaw function and occlusal behavior. Together, these two traditions describe complementary aspects of the same system—yet neither provides a mechanistic account of how central regulatory state and structural occlusal conditions interact dynamically through masticatory performance [9,17].

FORM proposes to integrate central regulatory state with peripheral jaw function—explaining occlusion not as an isolated structural variable but as a dynamic expression of the system’s central regulatory capacity, and in doing so, addressing the explanatory gap that neither tradition has resolved.

## 3. Theoretical Foundation of FORM

### 3.1. The Trigeminal System as a Regulatory Interface

The jaw is not simply a mechanical structure—it is a centrally regulated system. Jaw motor activity is coordinated by a trigeminally mediated central neural network that integrates inputs from the brainstem, hypothalamus, and limbic system to modulate masticatory function with broader physiological state. This architecture means that jaw behavior is continuously modulated by the same neural circuits that regulate arousal, stress response, and autonomic tone. It also means that masticatory activity is not merely an output of peripheral occlusal conditions—it is an expression of the central regulatory state in which the individual is operating [24,25].

This central integration provides the physiological pathway through which jaw function and autonomic state interact bidirectionally. Altered regulatory states—whether produced by psychological stress, sleep disruption, or heightened arousal—do not change occlusal morphology, but they do change how the masticatory system behaves. Conversely, masticatory activity itself can influence autonomic state through centrally mediated mechanisms, creating a functional loop between jaw use and physiological regulation.

### 3.2. Mastication, Autonomic Regulation, and Stress Buffering

Multiple lines of evidence demonstrate that mastication is not autonomically neutral. Higher masticatory performance has been associated with lower baseline sympathetic activity as indexed by heart rate variability (HRV) [26]. Chewing during acute stress exposure has been shown to reduce sympathetic nervous activation and stabilize cardiovascular responses, including the prevention of post-stress arrhythmias [27].

Animal experimental evidence extends these findings, demonstrating that rhythmic mastication modulates heart rate and stress-related brain activity through hypothalamic–autonomic mechanisms—centrally mediated pathways that link masticatory inputs with autonomic outputs through established neuroendocrine circuitry [24,28].

Human observational data is consistent with these experimental findings. Individuals with higher masticatory performance have been shown to exhibit lower psychosocial stress, higher HRV, lower salivary alpha-amylase—a sympathetic activity marker—and lower self-reported stress scores [29]. Critically, these associations were independent of tooth morphology, supporting the interpretation that masticatory performance reflects central regulatory capacity rather than favorable dental anatomy. A complementary finding links higher cardiac vagal tone with greater maximum occlusal force and more efficient eating behavior [29,30].

### 3.3. Breathing, Arousal, and Jaw Motor Control

Jaw function does not occur in isolation. It is embedded within a broader physiological context shaped by breathing pattern and arousal state. Breathing rhythm and pattern modulate emotional state and autonomic balance through established brainstem and limbic pathways, with slower, deeper breathing patterns associated with parasympathetic dominance and reduced arousal [31,32,33]. This means that habitual breathing behavior is not simply a respiratory variable; it is a significant variable shaping the regulatory environment in which masticatory function occurs.

Carbon dioxide (CO_2_) levels represent a relevant physiological variable within this context. Central chemosensory mechanisms are sensitive to CO_2_ and blood pH, and deviations from optimal CO_2_ ranges influence arousal, muscle tone, and motor control through brainstem regulatory circuits [33]. Habitual over-breathing—which reduces CO_2_ below optimal levels, inducing respiratory alkalosis and reducing ionized calcium—can increase peripheral nerve excitability, elevate sympathetic tone, and reduce sensorimotor adaptability, all without any change to occlusal morphology [33,34,35].

**Figure 1:**
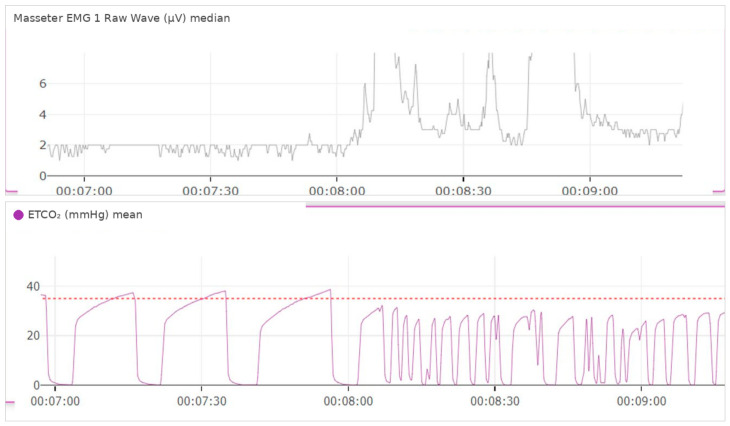
Clinical illustration of the real-time relationship between end-tidal CO_2_ (ETCO_2_, **lower** panel) and masseter electromyographic activity (EMG, **upper** panel) during capnometry-guided breathing training. When ETCO_2_ is maintained near the lower limit of the eucapnic range (~35 mmHg, dashed line), masseter EMG remains at baseline (approximately 2 μV), corresponding to the absence of reported pain. Following the onset of ventilatory instability, masseter EMG rises to approximately 6 μV, at which point pain returned. This figure is a clinical illustration consistent with the upstream regulatory influence of CO_2_ homeostasis on masticatory neuromuscular activity proposed within FORM. It does not represent experimental data and should not be interpreted as direct physiological measurement.

Within FORM, breathing is therefore recognized as a regulatory variable that may shape the neuromuscular environment of jaw function. Heightened arousal states—whether driven by stress, dysfunctional breathing, or autonomic dysregulation—are associated with increased muscle tension, reduced motor variability, and less efficient jaw movement. Calmer regulatory states, by contrast, support smoother and more adaptable masticatory function. This framework helps explain a clinically common but poorly theorized observation: jaw symptoms frequently worsen during periods of psychological or physiological stress even when occlusal relationships remain entirely unchanged, as illustrated in Figure 1.

Sleep bruxism provides an objective and well-characterized example of how central regulatory processes are expressed through the masticatory system. Polysomnographic studies demonstrate that rhythmic masticatory muscle activity (RMMA), the physiological signature of sleep bruxism, occurs in close temporal association with cortical micro-arousals [36] and is consistently preceded by coordinated autonomic and respiratory activation, including increases in heart rate, sympathetic tone, and respiratory amplitude [37]. These findings are consistent with the interpretation that bruxism is not a primary dental phenomenon, but a downstream motor expression embedded within an arousal-related physiological cascade.

Micro-arousals themselves are frequently triggered by respiratory instability, including airway obstruction and fluctuations in ventilatory control, linking sleep bruxism to the broader domain of sleep-disordered breathing [36]. Within this framework, alterations in CO_2_ homeostasis—particularly hypocapnia—may contribute to ventilatory instability and heightened arousal susceptibility through their effects on central chemosensitivity, neuronal excitability, and autonomic regulation [33,34,35]. Although hypocapnia does not directly induce bruxism, it may act as an upstream modulator of arousal dynamics, thereby indirectly increasing RMMA. This sequence—respiratory dysregulation, autonomic activation, arousal, and motor output—provides a concrete physiological example of the hierarchical relationships proposed within FORM, in which central regulatory state is associated with and precedes masticatory behavior.

### 3.4. Neuromuscular Efficiency and the Masticatory System

The jaw musculature is particularly sensitive to central regulatory state. Experimental studies demonstrate that acute mental stress induces autonomic activation, resulting in altered masseter muscle hemodynamics and increased temporalis electromyographic activity. These findings indicate that neuromuscular behavior in the masticatory system is centrally modulated and not solely explained by local mechanical factors such as occlusion or joint relationships [38].

This central sensitivity helps explain the characteristic features of low masticatory performance observed in stress-sensitive conditions. Slower jaw closing velocity, irregular chewing trajectories, increased muscle co-contraction, and guarded movement patterns are not primarily products of unfavorable occlusal morphology—they reflect a nervous system operating under elevated regulatory load, producing conservative, effort-intensive movement rather than efficient, predictive motor control.

Systematic review evidence suggests that biofeedback interventions targeting masticatory muscle activity can reduce muscle tone and bruxism frequency, with associated improvements in TMD-related pain in many cases. These effects are thought to arise from modulation of central neuromuscular regulation rather than direct occlusal intervention, although outcomes—particularly for pain and sleep bruxism—remain variable [39].

### 3.5. Convergence: Toward an Integrated Model

The four domains reviewed in this section—central neural integration, mastication-autonomic interaction, breathing and arousal regulation, and neuromuscular efficiency—are not independent lines of evidence. They converge on a shared set of principles: that jaw function reflects and influences central regulatory state, that occlusal behavior is shaped by the physiological environment in which it occurs, and that masticatory performance is a functionally meaningful indicator of the regulatory capacity of the system.

These convergent findings provide the empirical and mechanistic foundation for the Functional Occlusion Regulated Model (FORM), presented in the following section. FORM integrates these principles into a coherent clinical framework, proposing that occlusion is best understood not as a fixed structural variable but as a dynamic functional interface whose behavior and clinical significance depend on the central regulatory state of the individual.

### 3.6. Directional Evidence Supporting Regulatory State as an Upstream Influence on Masticatory Function

While the evidence base supporting FORM draws primarily from observational and correlational studies, several lines of investigation provide more direct support for the directional relationship FORM proposes—that central regulatory state is upstream of and influences functional jaw behavior. The most direct experimental evidence comes from animal studies comparing chewing diet versus powdered diet conditions. Rats fed a chow diet demonstrated significantly lower heart rate, higher high-frequency band power, and higher root mean square of successive differences in R-wave intervals (RMSSD)—established parasympathetic markers—relative to animals fed a powdered diet. Molecular analysis revealed concurrent downregulation of vasopressin, corticotropin-releasing hormone, and thyrotropin-releasing hormone in the chewing group, providing a mechanistic basis for the autonomic differences observed. These findings suggest that masticatory stimulation itself—independent of nutritional content—may upregulate parasympathetic tone through hypothalamic–autonomic pathways [28]. Complementary experimental evidence demonstrates that chewing during acute stress exposure reduces sympathetic nervous system activation and prevents post-stress cardiovascular arrhythmia in animal models, indicating that jaw function can modulate central regulatory state through centrally mediated pathways [27]. In human subjects, mental stress induces measurable changes in masticatory muscle hemodynamics through sympathetic nervous system activation, independent of occlusal morphology, consistent with the interpretation that central regulatory state is physiologically expressed through jaw muscle behavior via identifiable autonomic pathways [38]. The sympathetic nervous system provides modulatory innervation to skeletal muscle, influencing neuromuscular junction function, vascular regulation, and motor unit behavior. This pathway supports the concept that motor output is shaped by systemic regulatory factors, not local mechanical conditions alone [40].

The temporal relationship between autonomic state and masticatory performance has been examined in human cross-sectional studies. Baseline parasympathetic tone, measured before mastication, predicts subsequent maximum occlusal force in healthy individuals, a temporal sequence consistent with autonomic regulatory capacity as an upstream influence on functional jaw performance rather than a consequence of it [29]. Critically, the relationship is not merely binary but dose dependent. Individuals with higher masticatory ability demonstrate greater suppression of both autonomic stress markers (LF/HF ratio, salivary alpha-amylase) and subjective stress experience following psychosocial stress loading, compared to individuals with lower masticatory ability [29]. Studies examining chewing force confirm a dose–response: stronger chewing force produces greater suppression of salivary cortisol following mental stress [41,42]. The efficiency and quality of masticatory function—not simply its presence—is associated with the magnitude of autonomic benefit, consistent with FORM’s proposition that masticatory performance is a graded expression of underlying regulatory capacity. The number of chewing strokes has similarly been identified as the masticatory function-related factor most associated with stress relief, further supporting the relationship between functional quality and regulatory outcome [42]. At the systems level, experimental disruption of masticatory function through occlusal disharmony produces chronic stress and suppresses spatial learning in animal models, suggesting that functional compromise may propagate upstream to regulatory dysfunction and reflect the bidirectional nature of the masticatory-autonomic relationship [43]. Preserved masticatory function has been associated with maintained hippocampal structure and cognitive resilience, while sustained functional deficits produce cumulative regulatory and neurological consequences over time [44]. These findings are consistent with FORM’s core proposition that central regulatory state may represent a significant upstream influence on masticatory system function, rather than merely a correlate of it.

## 4. The Functional Occlusion Regulated Model (FORM)

### 4.1. Defining FORM

FORM proposes that occlusion is not a structural cause of jaw dysfunction but a functional output of the body’s regulatory state—organized within a hierarchy in which autonomic and neuromuscular regulation is proposed to shape masticatory performance, which in turn influences how structural occlusal conditions are expressed and clinically experienced. Within FORM, the clinical behavior of the masticatory system—how it performs, adapts, and responds to therapeutic intervention—is proposed to be influenced substantially by the central regulatory state of the individual rather than by the geometry of tooth contacts alone. Occlusal morphology provides the structural framework within which jaw function occurs, but the masticatory expression of those conditions is influenced by central regulatory capacity, autonomic balance, neuromuscular efficiency, and the physiological environment in which mastication takes place.

**Figure 2:**
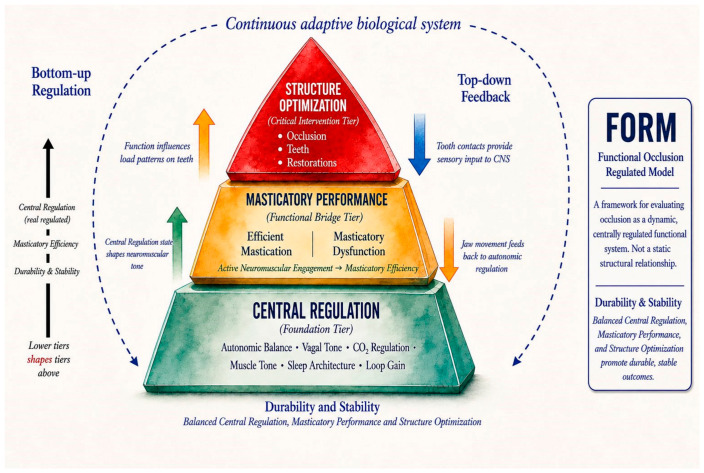
The Functional Occlusion Regulated Model (FORM) as a continuous adaptive biological system. Central Regulation (green, Foundation Tier) shapes Masticatory Performance (amber, Functional Bridge Tier), which influences structural expression through Structure Optimization (red, Clinical Intervention Tier). Bottom-up regulation and top-down sensory feedback reflect the bidirectional relationships between tiers. Durability and Stability are achieved when all three tiers are aligned.

FORM integrates three hierarchical domains: Central Regulation, Masticatory Performance, and Structure Optimization. These are not independent variables—they are organized in a regulatory hierarchy in which Central Regulation is proposed to shape Masticatory Performance, and Masticatory Performance influences how structural conditions are expressed and experienced. This hierarchy is the proposed integrative contribution that connects both traditional occlusal models and biopsychosocial frameworks within a unified physiological account (Figure 2).

### 4.2. The Three Tiers of FORM

Central regulation, as defined in the scientific literature, refers to CNS-mediated regulation of masticatory muscle activity through pathophysiological and psychosocial pathways, as distinguished from peripheral morphological influences such as occlusal discrepancy or condylar anatomy [45]. Lobbezoo and Naeije established this framework in 2001, concluding that bruxism is regulated primarily at the central level, with dopaminergic signaling, autonomic arousal, and psychological stress identified as principal mediating factors [45]. Lavigne and colleagues subsequently elaborated the neurobiological substrates of this regulation, demonstrating that brainstem structures that regulate rhythmic masticatory muscle activity share circuitry with those controlling respiration, sleep-state transitions, and autonomic output [46]. Within this framework, six regulatory components are recognized: autonomic balance, maintained through sympathetic and parasympathetic interaction [46,47]; vagal tone, indexed by heart rate variability and reflective of parasympathetic regulatory capacity [48]; CO_2_ regulation, mediated by central chemosensory mechanisms sensitive to arterial CO_2_ and blood pH [33]; muscle tone, modulated across sleep stages through monoaminergic and GABAergic pathways acting on brainstem motoneurons [46]; sleep architecture, which determines the arousal conditions under which masticatory motor events emerge [46]; and loop gain, which sets the stability of the ventilatory control system and thereby the susceptibility of the arousal threshold to respiratory perturbation [49]. Together, these components are proposed to constitute the physiological operating environment within which masticatory function occurs and through which central regulatory state is expressed in jaw behavior.

**Central Regulation** forms the foundation of the FORM hierarchy. It encompasses the autonomic balance, breathing pattern, arousal level, stress response, and baseline muscle tone that characterize an individual’s physiological operating environment at any given time. A well-regulated state, characterized by autonomic nervous system balance, efficient CO_2_ management, and low baseline arousal, supports smooth, adaptable, and efficient jaw function. A dysregulated state, characterized by sympathetic dominance, dysfunctional breathing, and elevated arousal, is associated with increased muscle tension, reduced motor adaptability, and guarded or parafunctional jaw behavior [26,31,33].

**Figure 3:**
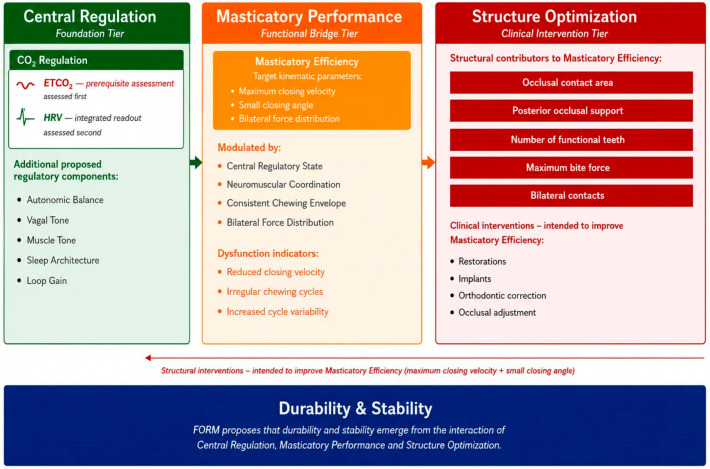
Operational schematic of FORM’s three-tier hierarchy. Central Regulation identifies CO_2_ Regulation as a proposed upstream contributor, with ETCO_2_ as the initial assessment and HRV as the integrated autonomic readout, followed by additional regulatory components. Masticatory Performance identifies target kinematic parameters, regulatory factors, and dysfunction indicators. Structure Optimization lists structural determinants and clinical interventions. See Sections The Role of CO_2_ Regulation Within FORM and 4.3 for operational rationale. This figure is an operational schematic and does not represent experimental data.

**Masticatory Performance** constitutes the middle tier, encompassing mastication, neuromuscular coordination, and load distribution during jaw use. Masticatory performance—how efficiently, smoothly, and comfortably an individual chews—serves as the key functional indicator within this tier [50]. High masticatory performance reflects a nervous system capable of predicting and guiding jaw closure efficiently, with coordinated muscle activity and smooth chewing trajectories [29]. Low masticatory performance reflects guarded movement, irregular trajectories, increased co-contraction, and elevated functional effort [50,51,52]. Critically, masticatory performance is influenced not only by structural occlusal conditions but also by the central regulatory state of the individual—the same structural occlusion may support different functional outcomes depending on regulatory context, a limitation not explained by traditional occlusal models [29]. Masticatory efficiency within FORM is defined operationally by two literature-supported kinematic parameters: maximum closing velocity and small closing angle [50]. These parameters represent the functional output of a well-regulated neuromuscular system—coordinated, efficient, and adaptive jaw closure. Dysfunction indicators—reduced closing velocity, irregular chewing trajectories, and increased cycle variability—reflect a nervous system operating under elevated regulatory load, producing guarded rather than efficient movement [50,51,52]. The five structural determinants of masticatory efficiency—occlusal contact area, posterior occlusal support, number of functional teeth, maximum bite force, and bilateral contacts—set the boundary conditions within which these kinematic parameters are achieved (Figure 3) [53,54]. Structural intervention addresses those boundary conditions. Regulatory intervention addresses the central state that influences whether the kinematic parameters are achieved within them.

**Structure** occupies the apex of the FORM pyramid. Not because it is the most important tier, but because it is the most distal from regulatory control. Structure Optimization encompasses occlusal relationships, tooth morphology, restorations, implants, and prosthetic components. Within FORM, structural variables define the operating envelope within which function must occur. The same structural occlusal condition may be well-tolerated in a highly regulated individual and poorly tolerated in a dysregulated one. This conditionality provides a mechanistic explanation for a gap that structural models have been unable to close why equivalent occlusal morphology produces such different clinical outcomes across patients [8,9].

#### The Role of CO_2_ Regulation Within FORM

Among the six components of central regulatory state, CO_2_ homeostasis is proposed as a transduction pathway through which psychological stress may become expressed as sustained physiological dysregulation. This position has direct consequences for how central regulatory state is defined, measured, and treated within FORM. The evidence converges from four independent research traditions: respiratory psychophysiology, sleep medicine, cerebrovascular neuroscience, and clinical anxiety research.

Psychological threat activates limbic–hypothalamic–brainstem respiratory circuits, increasing ventilation beyond metabolic need. This ventilatory increase is not metabolically driven. Masaoka and Homma demonstrated that anticipatory anxiety increased respiratory frequency and decreased end-tidal CO_2_ without any corresponding change in oxygen consumption, CO_2_ production, or heart rate, consistent with limbic modulation of respiratory drive independent of metabolic demand [55]. Van Diest and colleagues extended this to conditioned fear: differential fear conditioning produced a significant decrease in ETCO_2_ and shorter respiratory cycle time, with these changes extinguishing when the conditioned association was extinguished—establishing that hypocapnia respiratory responses can be learned, cued, and maintained through conditioning mechanisms independent of conscious anxiety [56]. In clinical populations, resting hypocapnia is not confined to acute panic episodes. Blechert and colleagues found that panic disorder patients exhibited significantly lower resting PCO_2_ and higher cardiovascular sympathetic activation than healthy controls, indicating that habitual hypocapnia may characterize a persistent physiological state in stress-sensitive individuals [57]. Together, these findings suggest that CO_2_ dysregulation may precede autonomic activation in stress-sensitive individuals.

Once hypocapnia is established, it activates a second pathway that amplifies and sustains dysregulation independent of the original psychological trigger. Central chemosensory neurons—specifically retrotrapezoid nucleus neurons sensitive to pH and PCO_2_—detect the fall in CO_2_ and generate brainstem arousal signals that increase sympathetic activation and sustain elevated regulatory load [33]. This chemoreceptor-driven arousal is a proposed mechanism by which a transient psychological state may produce a persistent physiological condition: arousal drives further overbreathing, which maintains hypocapnia, which continues to activate the chemosensory mechanism, which sustains arousal. The downstream consequences are measurable and multi-systemic: hypocapnia increases peripheral nerve and neuromuscular excitability through alkalosis-driven reduction in ionized calcium [34], impairs somatosensory processing and sensorimotor control through cerebral vasoconstriction and reduced perfusion [35], and sustains the autonomic imbalance that heart rate variability indexes [48].

The potential upstream role of CO_2_ relative to ANS activity in this regulatory sequence is examined by direct measurement. Uryga and colleagues applied a controlled breathing protocol across three respiratory rates in 61 healthy volunteers, simultaneously measuring end-tidal CO_2_, heart rate variability, baroreflex sensitivity, and cerebral blood flow velocity [58]. Using a linear mixed-effects model adjusted for both ETCO_2_ and respiratory rate, they found that cerebrovascular gain was primarily driven by respiratory parameters and ETCO_2_ directly, with ANS metrics having negligible independent contribution. ANS activity significantly modulated cerebral autoregulatory quality only after ETCO_2_ was statistically accounted for. This finding is consistent with CO_2_ exerting upstream effects on cerebrovascular regulation that are partially independent of ANS-mediated pathways, suggesting that HRV may reflect a regulatory output that is itself shaped by CO_2_ status.

Individual susceptibility to CO_2_-driven dysregulation is partly determined by loop gain—the gain of the ventilatory control system’s chemoreflex response to CO_2_ perturbations [49,59]. Elevated loop gain produces exaggerated ventilatory responses to CO_2_ fluctuations that amplify hypocapnia excursions and sustain arousal dysregulation. Messineo and colleagues demonstrated that loop gain during sleep can be predicted by waking breath-hold duration—a measure of CO_2_ tolerance—suggesting that loop gain is a stable waking trait reflecting individual CO_2_ sensitivity rather than a sleep-specific phenomenon [60]. Deacon-Diaz and colleagues demonstrated that loop gain measured during daytime wakefulness was significantly elevated in OSA patients and was not reduced by six weeks of CPAP treatment despite successful correction of the structural airway obstruction [61]. Waltz and colleagues confirmed this independence using partial coherence analysis: the CO_2_ pathway and the ANS pathway contribute as distinct regulatory inputs to cerebrovascular control, and structural airway correction left both pathways unaltered [62]. These findings are consistent with the conclusion that CO_2_ chemosensitivity functions as an independent regulatory variable.

In the context of the masticatory system, Figure 1 provides a clinical illustration of the CO_2_-neuromuscular relationship: when ETCO_2_ falls near the lower limit of the eucapnic range (~35 mmHg), masseter EMG rises and pain returns; when CO_2_ is maintained at threshold, neuromuscular activity remains at baseline, consistent with the regulatory sequence FORM proposes.

The proposed upstream role of CO_2_ within central regulatory state suggests a clinical measurement sequence. End-tidal CO_2_ at rest, measured via capnometry, is the initial assessment—it indicates whether hypocapnia may be contributing to the regulatory disturbance before any intervention is initiated. Short-term resting heart rate variability—particularly the high-frequency component and RMSSD, indexed per established standards [48]—then serves as the integrated readout of the resulting autonomic state. Meuret and colleagues demonstrated in a randomized controlled trial that capnometry-assisted breathing therapy targeting normalization of end-tidal pCO_2_ produced significant and sustained improvements in both respiratory physiology and symptom burden, with effects maintained at 12-month follow-up [63]. This is consistent with FORM’s proposed treatment sequence: CO_2_ normalization establishes the precondition for parasympathetic responsiveness; HRV biofeedback then consolidates and sustains the regulatory shift [32]. CO_2_ normalization is necessary but not exclusively sufficient—breathing retraining also operates through respiratory rhythm regularization and reduced interoceptive threat appraisal—the learned tendency to interpret hypocapnia-generated bodily sensations as signs of danger—and these mechanisms may contribute independently of CO_2_ correction alone. Alternative regulatory pathways may contribute similarly, and the relative importance of CO_2_ regulation compared with other components remains to be determined.

In clinical research, a minimum operationalization of central regulatory state within FORM would include resting ETCO_2_ and short-term resting HRV measured in that sequence—two partially independent but physiologically linked dimensions of the regulatory environment. No single measure fully represents the construct; their combination may provide diagnostic and mechanistic specificity that neither alone can offer. It should be noted that the proposed role of CO_2_ within this regulatory sequence operates at the level of habitual resting state rather than at the level of individual bruxism episode triggering. Suzuki and colleagues measured end-tidal CO_2_ immediately before and after RMMA onset during polysomnography and found no pre-RMMA change in ETCO_2_, concluding that acute peri-episode CO_2_ fluctuations have little influence on sleep bruxism genesis [64]. This finding is consistent with FORM’s proposed mechanism, which does not predict that CO_2_ shifts trigger individual RMMA episodes. Rather, FORM proposes that habitual hypocapnia elevates baseline arousal susceptibility and autonomic load over time, creating regulatory conditions in which RMMA is more likely to emerge—a different level of analysis than peri-episode respiratory chemistry. Several mechanistic links within this proposed sequence remain hypothetical and require prospective empirical validation before causal conclusions can be drawn.

### 4.3. Masticatory Performance as a Functional Bridge

High masticatory performance has been associated with favorable autonomic markers including higher HRV and lower sympathetic activity biomarkers, supporting the interpretation that high masticatory performance reflects central regulatory capacity rather than favorable tooth morphology alone [26]. Low masticatory performance—slower jaw movement, irregular trajectories, elevated co-contraction, and guarded function—is consistently observed in stress-sensitive conditions and parafunctional behaviors even when structural occlusion appears acceptable [38,42].

### 4.4. Occlusion as a Functional Boundary Condition

FORM neither dismisses occlusion as clinically irrelevant nor restores it to primary etiologic status. Instead, occlusion is reconceptualized as a functional boundary condition—the structural envelope within which regulated jaw function must operate. Meta-analytic evidence indicates that occlusal adjustment is associated with pain mitigation in some patients with TMD—yet individual treatment responses remain highly variable, with outcomes dependent on study quality, pain location, and patient characteristics, and the global evidence base remains weak due to methodological limitations across most included trials [9]. That a pooled benefit is detectable across trials does not resolve the variability problem—it sharpens it: if occlusal adjustment produces a measurable group-level effect yet fails to help a substantial proportion of individual patients, the question of what determines who responds becomes more clinically urgent, not less. FORM proposes that baseline central regulatory state is that moderating variable, which is the basis of Prediction 3. Occlusal intervention modifies the structural boundary condition and may, through improved masticatory performance, contribute to shifts in autonomic regulatory state via established masticatory-autonomic feedback pathways. Where regulatory dysregulation is the primary driver of symptoms, however, structural intervention alone may be insufficient without concurrent regulatory assessment and intervention.

### 4.5. Testable Predictions of FORM

FORM generates specific predictions that distinguish it from existing models and that are testable within the framework of current clinical and research methodology:

Prediction 1. Masticatory performance will correlate more strongly with autonomic markers (HRV, sympathetic activity biomarkers) than with measures of static occlusal morphology across patient populations. This prediction is testable in cross-sectional studies measuring validated masticatory performance (color-changeable chewing gum), short-term HRV recordings, and standardized occlusal examination in TMD and non-TMD populations, using multiple regression to compare the variance in masticatory performance explained by autonomic versus structural variables.

Prediction 2. Interventions that improve central regulatory state—including stress reduction, HRV biofeedback, and breathing retraining—will produce measurable improvements in masticatory performance independent of any occlusal modification. Randomized controlled trials would test Prediction 2 by assigning participants to regulatory-state intervention versus waitlist control, with masticatory performance and HRV as co-primary outcomes measured before and after intervention without any concurrent occlusal treatment.

Prediction 3. The response to occlusal intervention will be moderated by baseline central regulatory state: individuals with higher pre-treatment HRV and masticatory performance will show greater and more durable benefit from structural occlusal correction than those with lower regulatory capacity. Prediction 3 is amenable to testing by stratifying existing or prospective occlusal intervention trial populations by baseline HRV and masticatory performance quartile and comparing treatment response trajectories across strata at three, six, and twelve months.

Prediction 4. In stress-sensitive conditions such as TMD and bruxism, fluctuations in masticatory performance will track changes in central regulatory state more closely than changes in occlusal morphology over time. Longitudinal observational studies using repeated-measures assessment of HRV, masticatory performance, and standardized occlusal examination at monthly intervals over six to twelve months would test Prediction 4, with time-series analysis comparing the temporal coupling of autonomic and masticatory variables against occlusal morphology variables.

These predictions are falsifiable using clinical measurement tools already available—short-term HRV monitoring, validated masticatory performance assessments using color-changeable gum, and standardized occlusal examination protocols—and provide a specific, actionable research agenda consistent with a mechanistic regulatory framework.

## 5. Clinical Implications of FORM

### 5.1. Reframing Clinical Decision-Making

Within FORM, an occlusal finding is one input into a broader functional assessment that also considers the patient’s central regulatory state, masticatory performance, and adaptive capacity. In this framework, the clinical question shifts from “is the occlusion ideal?” to “is this patient’s functional system operating efficiently, and if not, what is the predominant limiting factor?”.

This reorientation does not eliminate structural occlusal assessment, it contextualizes it. A patient with reduced masticatory performance, elevated muscle tension, and compromised autonomic regulation presenting alongside an occlusal discrepancy may not benefit from occlusal correction alone if the underlying driver is regulatory rather than structural. The same occlusal discrepancy in a well-regulated patient with high masticatory performance may warrant a different clinical response. FORM provides a framework for making this distinction systematically rather than intuitively.

### 5.2. Interpreting Variable Treatment Responses

A patient who fails to respond to a well-executed occlusal appliance may not have a structural problem that has been insufficiently addressed. Within FORM, non-response may indicate that the predominant driver is regulatory—elevated autonomic arousal, dysfunctional breathing, or compromised neuromuscular efficiency. This interpretation is consistent with evidence that oral appliance therapy modifies sensory input and motor patterns but does not reliably alter the autonomic regulatory parameters that FORM proposes as key upstream influences on functional outcome [65].

### 5.3. TMD and Bruxism Through the FORM Lens

Temporomandibular disorders and bruxism represent clinical conditions in which the explanatory value of regulatory models becomes particularly apparent, as these conditions are highly sensitive to disruptions in autonomic, stress-related, and cognitive control systems [66].

Sleep bruxism illustrates this regulatory-to-motor pathway with clarity. Polysomnographic evidence demonstrates that RMMA occurs in close temporal association with cortical micro-arousals and is consistently preceded by autonomic and respiratory activation—increases in heart rate, sympathetic tone, and respiratory amplitude. This is consistent with bruxism episodes arising as downstream motor outputs within a centrally organized arousal cascade, rather than being initiated at the occlusal level [36,37]. Micro-arousals are frequently triggered by respiratory instability and sleep-disordered breathing, and upstream dysregulation of CO_2_ homeostasis may heighten arousal susceptibility through its effects on chemosensitivity and autonomic tone [35,36,37]. Within FORM, this sequence—respiratory dysregulation, autonomic activation, cortical arousal, and masticatory motor output—is what the regulatory hierarchy predicts: central state is associated with and precedes jaw behavior in a temporally consistent pattern.

FORM does not preclude occlusal intervention in TMD and bruxism management. Where structural conditions are genuinely limiting functional performance, structural correction may be warranted. The distinction FORM introduces is that this determination should be made in the context of a full regulatory and functional assessment, not based on structural findings alone. This is consistent with current evidence that conservative, reversible approaches should precede irreversible occlusal intervention, and it provides a clearer physiological rationale for that clinical recommendation [8].

The clinical applications presented in Section 5.4, Section 5.5, Section 5.6 and Section 5.7 represent theoretical extensions of FORM and have not been directly tested in clinical research. They are offered as hypothesis-generating frameworks for future investigation, not as evidence-supported clinical conclusions.

### 5.4. Implant Dentistry

Implant-supported restorations occasionally fail in ways that structural analysis cannot fully explain. Misch and colleagues proposed that occlusal overload may represent a more common source of implant complications than infection, including marginal bone loss and prosthetic failure, framing the problem as one requiring a force management approach to treatment planning [67]. However, Kim and colleagues noted that currently there is no evidence-based, implant-specific concept of occlusion, identifying bruxism and parafunctions as overloading factors without accounting for why those forces arise or how they are regulated [68]. Bruxism is a recognized risk factor for mechanical implant complications—a systematic review and meta-analysis found that bruxism increased the risk of prosthetic screw fracture 7-fold and implant fracture more than 16-fold compared to non-bruxing patients [69]. A second meta-analysis reported a pooled odds ratio of 4.68 for implant failure in patients with bruxism [70]. Within FORM, bruxism is a downstream motor expression of central regulatory dysregulation rather than a primary dental phenomenon. Osseointegrated implants additionally lack periodontal ligament mechanoreceptors, removing the principal proprioceptive feedback pathway that modulates occlusal loading. The central regulatory question—whether the patient’s autonomic state is contributing to neuromuscular hypertonicity that may amplify occlusal forces—is not captured by conventional structural implant assessment.

### 5.5. Orthodontic Treatment

Orthodontic treatment imposes sustained adaptive demands on the masticatory system by progressively altering occlusal relationships and jaw motor coordination over months to years. Patients with high regulatory capacity are better positioned to adapt smoothly to progressive occlusal change. Those with compromised regulatory capacity may experience greater functional disruption and symptom expression during treatment—not because of the orthodontic mechanics per se, but because their regulatory system is less able to accommodate the adaptive demands imposed. Within FORM, such presentations may reflect regulatory insufficiency rather than treatment-induced structural harm.

### 5.6. Restorative and Digital Dentistry

Contemporary restorative and digitally planned treatments—full-arch rehabilitations, vertical dimension changes, CAD/CAM-designed occlusal schemes—involve deliberate modification of structural occlusal conditions. Within FORM, the clinical success of these interventions depends not only on the accuracy of the structural design but on the patient’s functional and regulatory capacity to integrate the new occlusal scheme into efficient masticatory performance. FORM therefore supports the integration of functional and regulatory assessment into restorative treatment planning workflows, alongside conventional structural occlusal assessment.

### 5.7. Practical Clinical Proxies for Regulatory State

A practical question raised by FORM is how clinicians can assess central regulatory state and masticatory performance in routine practice. FORM identifies two instrument-based measures applicable in clinical settings: end-tidal CO_2_ (ETCO_2_), assessed via capnometry, and heart rate variability (HRV), assessed via short-term electrocardiographic recording. Both were used in the published case report that provides an illustrative clinical observation consistent with FORM’s regulatory framework, where a CapnoTrainer device provided simultaneous real-time ETCO_2_ and HRV monitoring during resonance frequency breathing training [71,72]. As wearable HRV monitoring and digital masticatory performance assessment become more widely available, more precise regulatory and functional data will increasingly be accessible within dental clinical settings.

## 6. Limitations and Future Directions

FORM is a theoretical framework, not a tested clinical protocol. Its limitations are real and worth stating plainly.

First, the evidence supporting FORM is drawn from studies not designed to test it directly. The relationships between central regulatory state, masticatory performance, and structural behavior are supported by converging observational, experimental, and cross-sectional evidence—but FORM as a complete hierarchy has not been prospectively validated. That validation requires clinical studies designed specifically around its constructs, which do not yet exist.

Second, while instrument-based assessment of central regulatory state—specifically resting ETCO_2_ via capnometry and short-term HRV via electrocardiographic recording—is clinically accessible and has been applied in the published case observation consistent with this framework [71], prospective validation of this measurement approach in dental populations has not yet been conducted. Establishing standardized protocols and clinically meaningful thresholds for these measures in TMD and bruxism populations remains a necessary next step.

Third, the relative weighting of FORM’s three tiers—how much Central Regulation, Masticatory Performance, and Structure Optimization each contribute in a given patient—has not been quantified. Different patients will present with different limiting factors, and the model’s hierarchy should be understood as a general organizing principle rather than a fixed formula.

Fourth, the preliminary clinical observation presented consists of a single published case report by the author who also proposes FORM [71]. While the outcome is consistent with FORM’s regulatory hierarchy and has been independently peer-reviewed, a single case cannot establish generalizability. Independent replication across diverse clinical populations is a necessary next step before FORM’s predictions can be considered supported by clinical evidence rather than clinical observation.

## 7. Conclusions

The long-standing clinical controversy surrounding occlusion, temporomandibular disorders, and bruxism persists not because the evidence is absent, but because existing frameworks have been unable to integrate it into a coherent, physiologically grounded model that connects central regulatory state to functional jaw behavior in clinically actionable terms.

FORM is offered not as a replacement for existing frameworks but as an integrative advance: a model that gives clinicians a biologically grounded, physiologically specific, and practically applicable way to think about occlusion as a dynamic functional system rather than a static structural ideal. FORM proposes that individual differences in central regulatory state may help explain the clinical variability that structural assessment alone cannot explain.

## Data Availability

No new data were generated or analyzed in this study.
